# Genetics-guided screening and biological validation identify zeylenol as a promising EGFR inhibitor in glioma

**DOI:** 10.3389/fgene.2026.1822595

**Published:** 2026-04-29

**Authors:** Lin Pan, Yifan Li, Leyan Chen, Wenzhuo Yang, Haiqin Gao, Yuxin Hou, Shengxuan Liu, Zibing Zhao, Xinhui Wang, Hongyu Chen, Dong Wang

**Affiliations:** 1 Guangdong Sanjiu Brain Hospital, Guangzhou, China; 2 Department of Neurology, Neuroscience Research Center, The First Hospital of Jilin University, Changchun, China; 3 Department of Neurosurgery, State Key Laboratory of Oncology in South China, Collaborative Innovation Center for Cancer Medicine, Sun Yat-sen University Cancer Center, Guangzhou, China; 4 ZhongShan School of Medicine, Sun Yat-Sen University, Guangzhou, China; 5 Department of Oncology, First People’s Hospital of Xinxiang & The Fifth Affiliated Hospital of Xinxiang Medical College, Xinxiang, China

**Keywords:** drug repurposing, EGFR, glioma, mendelian randomization, natural inhibitors

## Abstract

**Introduction:**

To overcome the high attrition rates in glioma drug discovery, this study established a systematic “Genetics-to-Drug” pipeline aimed at identifying potential therapeutic targets for glioma through causal inference and discovering potent natural inhibitors.

**Methods:**

We initiated the study with a drug-target Mendelian Randomization (MR) analysis, leveraging quantitative trait loci (QTL) and large-scale genome-wide association studies (GWAS) data for all glioma, glioblastoma (GBM), and non-GBM subtypes. Based on the identified targets, we performed a virtual screening of 17,931 ZINC15 compounds utilizing LibDock, absorption, distribution, metabolism, and excretion (ADME) profiling, toxicity prediction by komputer assisted technology (TOPKAT), CDOCKER, and molecular dynamics (MD) simulations. The binding affinity of selected candidates was confirmed via biolayer interferometry (BLI), and the therapeutic efficacy of the identified small molecules were biologically validated through *in vitro* assays on glioma cells.

**Results:**

The druggable MR screening of 140 genes identified EGFR as a key causal target across all glioma subtypes (GBM, non-GBM, and all glioma). Through a series of computer-aided techniques, two promising lead compounds, tremulacin (ZINC000004098458) and zeylenol (ZINC000229763735), were prioritized. Subsequent BLI assays validated a direct physical interaction between zeylenol and EGFR, and biological validation demonstrated that zeylenol treatment significantly suppressed GBM cell proliferation and migration while promoting apoptosis, supporting its therapeutic potential as a novel EGFR inhibitor.

**Discussion:**

This study identifies EGFR as a causally relevant therapeutic target in glioma and highlights tremulacin and zeylenol as promising natural EGFR inhibitors, warranting further preclinical development of these compounds as novel glioma therapeutics.

## 1 Introduction

Brain tumors are associated with disproportionately high mortality rates, reaching approximately 30% in children and 20% in adults (McNeill, 2016). Among these, gliomas represent the most common primary central nervous system (CNS) tumors (Zhao et al., 2021). The prognosis remains particularly dismal for older populations. For instance, patients aged 55 to 64 face a 5-year survival rate of merely 4.6% (Colquhoun, 2017). The current standard of care involves maximal surgical resection followed by radiotherapy and chemotherapy, primarily using temozolomide (TMZ) or the PCV regimen (Stupp et al., 2005). However, the therapeutic efficacy of these agents is severely limited by the unique anatomical and physiological properties of the CNS. The blood-brain barrier (BBB) restricts the delivery of most chemotherapeutic agents, while the highly invasive nature of glioma cells frequently leads to recurrence (Liu X. et al., 2023; Norden and Wen, 2006). Consequently, there is an urgent unmet need to identify robust therapeutic targets and develop novel agents with improved efficacy.

The high attrition rate in clinical trials constitutes a primary bottleneck in glioma drug development. Recently, druggable Mendelian randomization (MR) has emerged as a transformative strategy for target discovery. By utilizing genetic variants as instrumental variables (IVs) to proxy gene expression or protein levels, MR allows researchers to systematically screen and prioritize druggable genes before initiating costly drug development efforts (Gaziano et al., 2021; Finan et al., 2017). While this genomic approach has successfully identified targets for complex diseases like cerebrovascular and cardiometabolic disorders (Yang et al., 2024; Ye et al., 2025), its application in prioritizing therapeutic targets specifically for glioma remains largely unexplored.

Natural products have historically served as a rich source of scaffolds for drug discovery due to their high structural diversity and relatively low toxicity compared to synthetic counterparts. Indeed, numerous natural compounds, such as Vitexin and Scutellarin, have been successfully optimized into clinical candidates (Akter et al., 2021). However, traditional wet-lab screening of natural libraries is time-consuming and resource-intensive. To accelerate this process, structure-based virtual screening (SBVS) integrated with molecular dynamics (MD) simulations offers a powerful solution (Lionta et al., 2014). By computationally filtering vast databases like ZINC15 (Irwin and Shoichet, 2005), researchers can efficiently identify natural compounds with optimal binding affinities and pharmacokinetic profiles, bridging the gap between a genetic target and a physical drug candidate.

Motivated by these challenges, the present study aimed to establish a systematic “Genetics-to-Drug” pipeline for glioma therapy. First, we utilized a druggable MR framework to identify causal therapeutic targets for glioma. Second, based on the genetically prioritized target, we performed structure-based virtual screening and biolayer interferometry (BLI) validation to discover novel natural inhibitors. Finally, we conducted *in vitro* cellular assays to validate the anti-glioma efficacy of the identified candidate compounds.

## 2 Materials and methods

### 2.1 Identification of target genes

To explore the causal impact of gene levels on glioma risk, we first identified a set of biologically relevant and potentially druggable genes. We specifically curated 140 candidate genes from the database of drug targets, prioritizing those with established roles in gliomagenesis. These genes were categorized into five functional classes based on their established roles in gliomagenesis and targeted therapy development: cell cycle regulation, RTK signaling pathway, MAPK signaling pathway, epigenetic regulation, and immune checkpoint modulation (Supplementary Table S1).

### 2.2 Data sources and selection of genetic instruments

To construct genetic instruments proxying gene expression and protein levels, we extracted significant cis-expression quantitative trait loci (eQTLs) and cis-protein quantitative trait loci (pQTLs) from large-scale public datasets. For gene expression, cis-eQTLs were obtained from both blood (eQTLGen Consortium, https://www.eqtlgen.org/) (Võsa et al., 2021) and brain tissues (PsychENCODE Consortium, http://resource.psychencode.org/) (Wang et al., 2018). For protein abundance, cis-pQTLs were sourced exclusively from blood tissue datasets, including the deCODE study (N = 35,559) (Ferkingstad et al., 2021) and the INTERVAL study (N = 3,301) (Sun et al., 2018).

Outcome data for glioma and its subtypes were acquired from the largest available genome-wide association study (GWAS) meta-analysis of European ancestry, comprising 6,183 glioblastoma (GBM) cases, 5,820 non-GBM cases, and 18,169 controls (Melin et al., 2017). The detailed information was presented in Supplementary Table S2. To ensure instrument validity, all genetic variants were required to reach genome-wide significance (*P* < 5 × 10^−8^) and be located within ±1 Mb of the target gene (cis-acting). Independent SNPs were identified via linkage disequilibrium (LD) clumping (*r* ^2^ < 0.01, 1,000 kb window). To avoid sample overlap, all QTL and GWAS datasets were derived from independent cohorts. Exposure and outcome datasets were harmonized to ensure consistent effect allele orientation before causal estimation.

### 2.3 Mendelian randomization framework

The MR analysis was performed based on the number of available IVs. For exposures with a single valid SNP, the Wald ratio method was utilized as the primary analysis (Hemani et al., 2018). For exposures with multiple IVs, the inverse-variance weighted (IVW) method under a multiplicative random-effects model was employed (Liu Y. et al., 2023; Zhong et al., 2023). Sensitivity analyses were conducted using MR-Egger regression and the weighted median method to assess the robustness of the causal estimates (Burgess and Thompson, 2017). Associations that passed the Bonferroni correction (*P* < 0.05/140 = 3.57 × 10^−4^) were considered statistically significant. All MR analyses were implemented in R version 4.3.2 using the TwoSampleMR package.

### 2.4 Structure-based virtual screening through LibDock, absorption, distribution, metabolism, and excretion (ADME) and toxicity prediction by komputer assisted technology (TOPKAT)

Virtual screening was performed using Discovery Studio 2019 (Zhang et al., 2023; Zhong et al., 2022). A library of 17,931 natural, purchasable compounds was retrieved from the ZINC15 database. The crystal structure of the EGFR kinase domain complexed with the inhibitor TAK-285 (PDB ID: 3POZ, resolution 1.50 Å) was retrieved from the Protein Data Bank. This structure was selected due to its high crystallographic resolution and the presence of a co-crystallized ligand bound to the ATP-binding pocket, which provides a reliable structural reference for defining the docking site. The ligand-binding pocket was defined based on the reference ligand TAK-285. Compounds were docked using the LibDock algorithm (Katharine Holloway et al., 2007) and ranked by LibDock score. Top-ranked candidates underwent pharmacological profiling using the ADME module to predict aqueous solubility, blood-brain barrier (BBB) penetration, CYP2D6 inhibition, hepatotoxicity, absorption, and plasma protein binding (PPB). Safety profiles were assessed using the TOPKAT module, evaluating carcinogenicity (NTP classification), mutagenicity, and developmental toxicity potential (DTP).

### 2.5 Molecular docking and pharmacophore prediction

Molecular docking was performed using the CDOCKER module in Discovery Studio 2019 (Wu et al., 2003). The EGFR structure (PDB ID: 3POZ) was prepared by removing water molecules, adding hydrogen atoms, and performing energy minimization using the CHARMm36 force field. The binding site was defined as a sphere with a 16 Å radius centered on the co-crystallized ligand TAK-285. CDOCKER was used to dock flexible ligands into the rigid receptor, followed by energy minimization to refine the binding poses (Yang et al., 2022). The optimal binding conformations were selected based on interaction energy and docking score. The docking interactions were visualized using Schrödinger software. Pharmacophore features were subsequently identified based on the key interactions between the ligand and the active site residues.

### 2.6 Molecular dynamics simulation

MD simulations were performed using Discovery Studio 2019 with the CHARMm force field (Yang et al., 2023). The system underwent energy minimization using 500 steps of steepest descent followed by 500 steps of conjugate gradient algorithms (Zhao et al., 2022). Subsequently, the system was heated from 50 to 300 K over 2 ps and equilibrated for 5 ps. The production trajectory was generated for 150 ps at 300 K and 1 atm, utilizing a time step of 1 fs. We applied the Particle Mesh Ewald (PME) method for long-range electrostatics and the LINCS algorithm to constrain hydrogen-containing bonds. Structural stability was assessed by monitoring the root mean square deviation (RMSD) and potential energy (Zhong et al., 2021).

### 2.7 Biolayer interferometry

BLI assays were performed to characterize the direct binding interactions between zeylenol and the EGFR protein (Desai et al., 2019). Recombinant EGFR protein (TargetMol) was diluted to 20 μg/mL in PBST buffer and immobilized onto Ni-NTA biosensors (Sartorius). The assay procedure consisted of four steps: initial baseline (60 s), protein loading (60 s), association (100 s), and dissociation (200 s). The equilibrium dissociation constant (KD) was determined by testing the compounds at concentrations ranging from 50 to 400 μM. Sensorgrams were globally fitted to a 1:1 binding model using BLItz Pro v1.1.0.28 software.

### 2.8 Cell culture and reagents

The LN18 human GBM cell line was acquired from the Institute of Biochemistry and Cell Biology (Shanghai, China). Cultures were grown in Dulbecco’s Modified Eagle’s Medium (DMEM; Gibco) enriched with 10% fetal bovine serum (FBS; Gibco) under standard conditions (37 °C, 5% CO_2_, humidified atmosphere). For experimental use, zeylenol stock solutions were prepared in dimethyl sulfoxide (DMSO) and further diluted with the growth medium to achieve the specified working concentrations.

### 2.9 Cell viability and migration assays

The cell viability of LN18 cells was determined via the Cell Counting Kit-8 (CCK-8; DOJINDO, Japan). Briefly, cells were plated at a density of 5 × 10^3^ per well in 96-well plates and allowed to adhere overnight. Subsequently, cultures were exposed to graded doses of zeylenol for 24 or 48 h. Absorbance was recorded at 450 nm using a microplate reader following the addition of CCK-8 solution.

To investigate cell migration, we conducted wound-healing assays. LN18 cells were grown in 24-well plates until reaching ∼90% confluence. A scratch was manually induced using a sterile 10-μL pipette tip, followed by PBS washing to eliminate debris. The monolayers were then maintained in serum-free DMEM supplemented with zeylenol or 0.1% DMSO. Photomicrographs were taken at 0, 24, and 48 h, and migration distance was calculated using ImageJ software based on six random fields.

### 2.10 Apoptosis analysis via flow cytometry

To evaluate cell apoptosis, we employed an Annexin V-FITC/Propidium Iodide (PI) staining kit. LN18 cells were seeded at a density of 2 × 10^5^ cells per well and incubated overnight. Subsequently, the cells were exposed to varying concentrations of zeylenol or 0.1% DMSO (control) for 48 h. Following treatment, cells were detached using Accutase (Sigma-Aldrich), rinsed twice with ice-cold PBS, and suspended in binding buffer. Double-staining was performed with Annexin V-FITC and PI for 15 min at room temperature. Data acquisition was conducted using a BD Biosciences flow cytometer and analyzed via FACSDiva software (Version 6.2).

### 2.11 Statistical analysis

Statistical analyses were performed using SPSS 21.0, while figures were generated with GraphPad Prism 8. Quantitative results are expressed as mean ± standard deviation (SD) from at least three independent experiments. Comparisons between two groups were performed using two-tailed unpaired Student's t-tests, while one-way analysis of variance (ANOVA) followed by Tukey’s *post hoc* test was applied for comparisons among multiple groups. A *P* value <0.05 was considered statistically significant. Statistical significance was indicated as *P < 0.05, **P < 0.01, and ***P < 0.001.

## 3 Results

### 3.1 Druggable MR screening identifies EGFR as a key causal target for glioma

**FIGURE 1:**
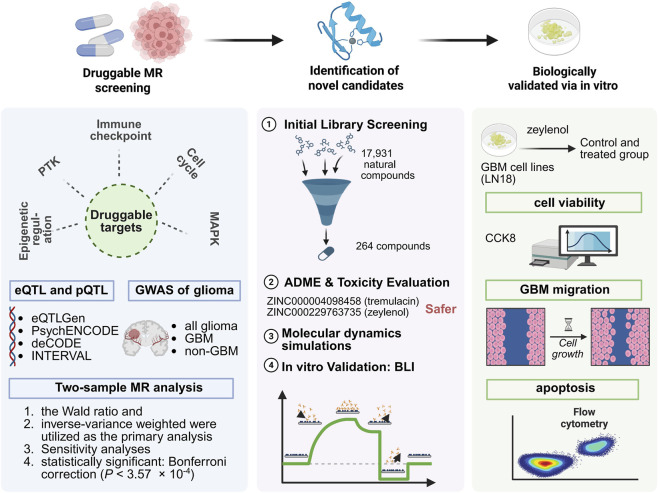
Flow chart of study design and analytical approach. Abbreviations: MR, Mendelian randomization; eQTL, expression quantitative trait loci; pQTL, protein quantitative trait loci; GWAS, genome-wide association study. Created with BioRender.com.

**FIGURE 2:**
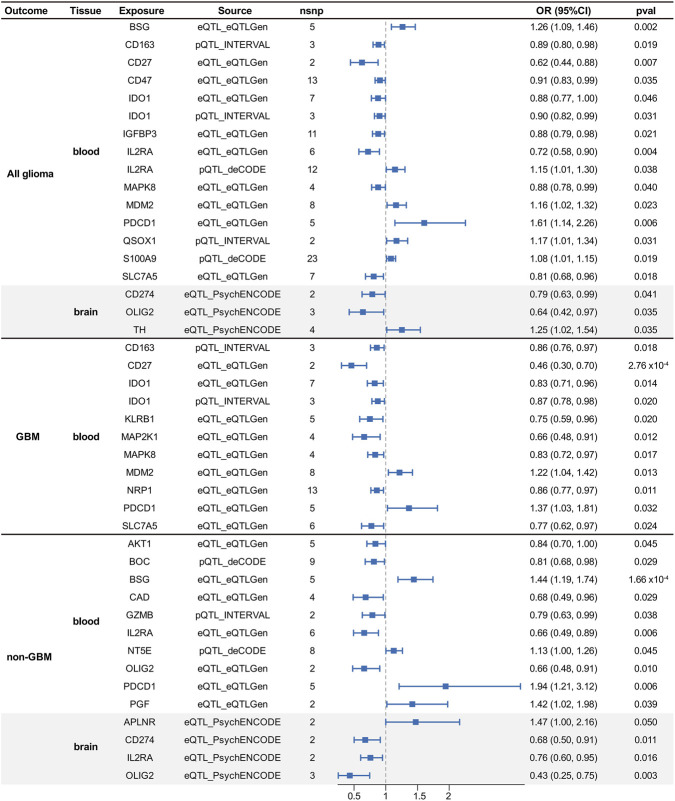
MR analysis results of eQTLs and pQTLs with glioma risk in blood and brain tissues. The forest plot presents causal estimates derived from the IVW method only. Results obtained using the Wald ratio are reported in the corresponding Supplementary Tables. Data are expressed as Odds Ratios (OR) with 95% Confidence Intervals (CI). An OR > 1 indicates a risk factor, while an OR < 1 indicates a protective factor. *P*-values <0.05 are considered nominally significant. Bonferroni-corrected *P*-values <3.57 × 10^−4^ are considered statistically significant. Abbreviations: nSNP, number of single nucleotide polymorphisms.

The overall design of this study is presented in Figure 1. To systematically identify therapeutic targets for glioma, we prioritized 140 potentially druggable genes spanning five key tumorigenic pathways: RTK signaling, immune checkpoints, MAPK signaling, epigenetic regulation, and cell cycle (Supplementary Table S1). By integrating both expression (eQTL) and protein (pQTL) data, we robustly evaluated the causal associations of these targets with glioma risk. After Bonferroni correction (*P* < 3.57 × 10^−4^), we identified two genes (CD27 and EGFR) related to GBM, three genes (IDH1, BSG and EGFR) related to non-GBM, and two genes (IDH1 and EGFR) related to all glioma (Figure 2; Supplementary Tables S3-S6). Among these genes, EGFR demonstrated the most robust and consistent causal association with all glioma subtypes. Specifically, genetically predicted higher EGFR expression in brain was significantly associated with a lower risk of all glioma (odds ratio [OR] = 0.42, 95% CI: 0.36–0.50, *P* = 1.07 × 10^−25^), GBM (OR = 0.31, 95% CI: 0.25–0.38, *P* = 2.89 × 10^−31^), and non-GBM subtypes (OR = 0.56, 95% CI: 0.45–0.69, *P* = 1.60 × 10^−7^). Although the direction of association suggests a protective effect at the population level, EGFR remains a biologically relevant therapeutic target due to its frequent somatic amplification and mutation within tumor tissues. Therefore, subsequent analyses focused on the identification of potential EGFR inhibitors using structure-based computational screening.

### 3.2 Identification of drug candidates targeting EGFR via multi-stage virtual screening

**FIGURE 3:**
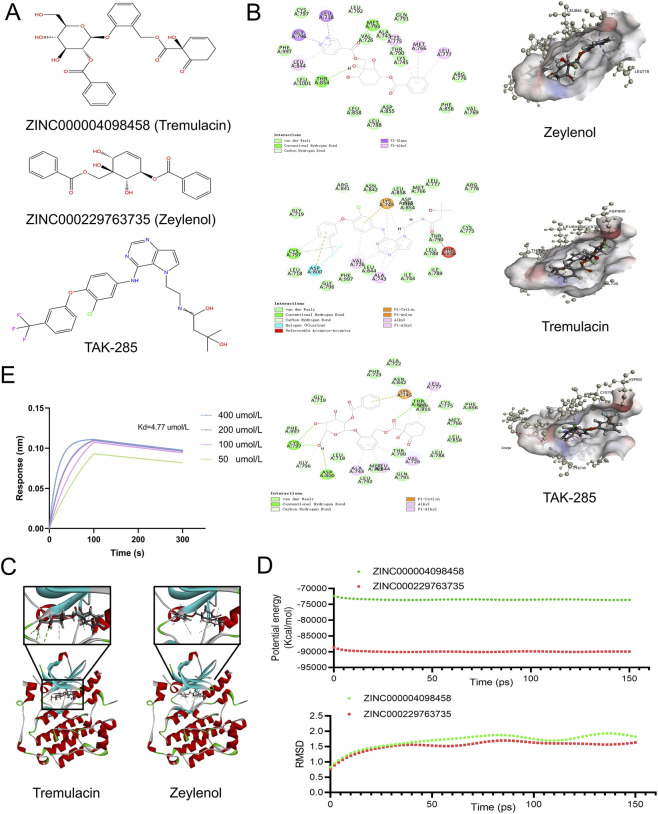
The results of virtual screening and BLI validation for the identified small molecule inhibitors. **(A)** The 2D structures of TAK-285 and novel compounds were selected from virtual screening. **(B,C)** Schematic drawing of interactions between ligands and EGFR. **(D)** Results of molecular dynamics simulation of the compounds tremulacin (ZINC000004098458) and zeylenol (ZINC000229763735). **(E)** KD determination of zeylenol and EGFR using BLI.

To translate the genetic findings into therapeutic interventions, we performed a hierarchical virtual screening of 17,931 natural compounds from the ZINC15 database, using the established inhibitor TAK-285 as a reference pharmacophore. First, based on ligand-binding pocket characteristics, 264 compounds were prioritized for exhibiting pharmacologic scores superior to TAK-285. The top 30 scored compounds are listed in Supplementary Table S7. Subsequently, we applied stringent ADME and toxicity filters to ensure clinical translatability. While TAK-285 presented certain limitations in hepatotoxicity and metabolic stability, our screening identified two lead candidates, tremulacin (ZINC000004098458) and zeylenol (ZINC000229763735), which displayed optimal drug-like properties. As detailed in Table 1 and Supplementary Table S8, these compounds exhibited excellent aqueous solubility and were predicted to be non-inhibitors of CYP2D6, minimizing drug-drug interaction risks. Crucially, toxicity prediction revealed that both candidates exhibited generally more favorable predicted toxicity profiles than TAK-285, particularly in terms of mutagenicity. (Supplementary Table S9), making them safer candidates for further development. The chemical structures of TAK-285, tremulacin (ZINC000004098458) and zeylenol (ZINC000229763735) are shown in Figure 3A.

**TABLE 1:** Absorption, distribution, metabolism, and excretion properties of compounds.

Compounds	Solubility level	BBB level	CYP2D6	Hepatotoxicity	Absorption level	PPB level
Tremulacin	3	4	0	0	3	0
Zeylenol	3	4	0	0	0	1
TAK-285	1	4	0	1	2	1

BBB, blood-brain barrier; CYP2D6, cytochrome P-450 2D6; PPB, plasma protein binding.

Aqueous-solubility level: 0, extremely low; 1, very low, but possible; 2, low; 3, good.

BBB, level: 0, very high penetrant; 1, high; 2, medium; 3, low; 4, undefined.

CYP2D6 level: 0, non-inhibitor; 1, inhibitor.

Hepatotoxicity: 0, non-toxic; 1, toxic.

Human-intestinal absorption level: 0, good; 1, moderate; 2, poor; 3, very poor.

PPB, level: 0, weak; 1, strong.

### 3.3 MD simulation and BLI confirm stable EGFR-ligand interactions

To elucidate the interaction mechanism and validate the physical binding affinity, we combined molecular docking, MD simulations, and BLI assays. The CDOCKER analysis revealed that both tremulacin (ZINC000004098458) and zeylenol (ZINC000229763735) possessed lower potential energies and interaction energies than TAK-285, indicating a tighter and more thermodynamically favorable binding to the EGFR (Supplementary Table S10). Structural analysis demonstrated that these compounds fitted precisely into the binding pocket through extensive hydrogen bond networks and hydrophobic interactions (Figures 3B,C). Notably, tremulacin (ZINC000004098458) formed five critical hydrogen bonds, including interactions with key residues CYS797 and ASP800, which were essential for kinase inhibition. The hydrophobic interactions with the compound involved LEU777, VAL726, ALA743, and LEU844 of 3POZ. Similarly, zeylenol (ZINC000229763735) established a robust interaction network involving three hydrogen bonds and six hydrophobic contacts. Specifically, hydrogen bonds were observed between the compound’s O1 atom and residues MET793 (HN) and LEU792 (HA), as well as between the H31 atom and THR854 (OG1). Furthermore, the complex was stabilized by hydrophobic interactions involving residues LEU718, GLY796, MET766, CYS775, LEU777, and LEU844 (Supplementary Table S11).

Furthermore, 150 ps MD simulations confirmed the dynamic stability of these complexes (Figure 3D). The potential energy of both systems rapidly reached equilibrium at the early stage of the simulation and remained stable throughout the 150 ps trajectory, indicating that the systems were energetically well equilibrated. The RMSD analysis showed an initial increase during the first ∼20 ps, followed by stabilization with only minor fluctuations for the remainder of the simulation, suggesting that both ligand-EGFR complexes maintained stable conformations within the binding pocket under physiological-like conditions. Collectively, these computational results further support the favorable binding stability of the identified ZINC compounds with EGFR.

To experimentally verify these *in silico* predictions, BLI assays were performed to measure the direct binding affinity between the candidates and the recombinant EGFR protein. The real-time binding sensorgrams demonstrated that zeylenol bound to EGFR in a dose-dependent manner across a concentration range of 50–400 μM, exhibiting fast association and stable dissociation kinetics (Figure 3E). The equilibrium dissociation constant was calculated to be 4.77 μM, indicating a direct physical interaction. These biophysical data strongly support the computational findings that zeylenol acts as a direct binder of EGFR.

### 3.4 Biological validation: Zeylenol treatment impairs GBM cell proliferation and migration and promotes apoptosis

**FIGURE 4:**
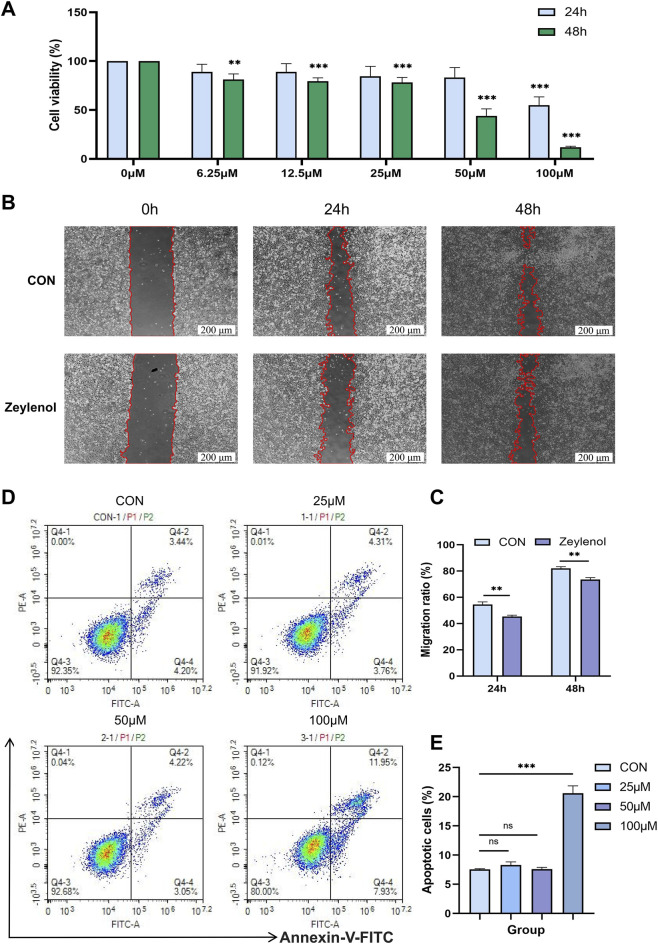
Zeylenol inhibited the proliferation and migration of glioma cells and promoted apoptosis. **(A)** Cell viability of LN18 cells was assessed via CCK-8 assay following treatment with indicated concentrations of zeylenol or vehicle (DMSO) for 24 and 48 h. **(B)** Representative images of the wound-healing assay showing the migration of LN18 cells treated with control or 100 μM zeylenol at 0, 24, and 48 h. Scale bar = 200 μm. **(C)** Quantification of the migration rates from the wound-healing assay. **(D,E)** Flow cytometric analysis of apoptosis. LN18 cells were treated with vehicle or graded concentrations of zeylenol for 48 h prior to analysis. Data are presented as representative results or mean ± SD from three independent experiments. *P < 0.05, **P < 0.01, ***P < 0.001.

To experimentally validate the therapeutic potential of zeylenol predicted by our computational findings, we conducted *in vitro* assays on the LN18 GBM cell line. Cell viability was first assessed using the CCK-8 assay following incubation with zeylenol at concentrations ranging from 0 to 100 μM for 24 and 48 h. The results demonstrated that zeylenol treatment significantly inhibited GBM cell proliferation in a dose- and time-dependent manner (Figure 4A). Notably, the most potent cytotoxic effect was observed at the 100 μM concentration at 48 h.

Given the highly invasive nature of GBM, we further evaluated the impact of zeylenol on cell motility using a wound-healing assay. While the control group exhibited rapid migration with near-complete gap closure by 48 h, the group treated with 100 μM zeylenol showed a marked suppression of wound healing capacity at both 24 h and 48 h intervals (Figures 4B,C). This indicates that zeylenol effectively attenuates the migratory potential of GBM cells.

To investigate whether the growth inhibition was associated with cell death, we performed flow cytometry analysis. As shown in Figure 4D, zeylenol treatment led to a dose-dependent trend toward increased apoptosis. Quantitative analysis confirmed a statistically significant increase in the apoptotic rate in zeylenol-treated cells compared to the control group, particularly at the 100 μM concentration (Figure 4E). Collectively, these biological validations corroborate our computational predictions, demonstrating that zeylenol significantly suppresses GBM progression by inhibiting proliferation and migration while promoting apoptosis.

## 4 Discussion

In the present study, we established a systematic “Genetics-to-Drug” pipeline by integrating MR, drug screening, and biological validation. Our MR analysis robustly identified EGFR as a causal target across all glioma subtypes. Subsequent structural screening of the ZINC15 database prioritized two natural compounds, tremulacin and zeylenol. Crucially, we provided direct biophysical evidence via BLI that zeylenol binds EGFR with micromolar affinity (KD = 4.77 μM). Finally, *in vitro* assays validated that zeylenol significantly reduced LN18 cell viability and migration while promoting apoptosis, providing compelling evidence for its potential as a novel therapeutic agent.

Human epidermal growth factor receptor gene family consists of four members, which are ERBB1, ERBB2, ERBB3, ERBB4. They belong to the ErbB family of proteins (Roskoski, 2019). EGFR can connect extracellular signals with crucial physiological processes, such as cell survival, growth, proliferation, and differentiation (Wu and Zhang, 2020). In glioma, EGFR is one of the most frequently altered oncogenic drivers. Amplification and activating mutations, particularly the constitutively active EGFRvIII variant, are commonly observed in GBM and lead to persistent activation of downstream pathways such as PI3K/AKT and MAPK (An et al., 2018; Pelloski et al., 2007; Deschênes-Simard et al., 2014). This aberrant EGFR signaling promotes tumorigenesis and progression in GBM. Our study was motivated by genetic evidence that underscores the pivotal role of EGFR in glioma. Using MR, we observed that higher genetically predicted EGFR expression in brain tissue was associated with a reduced risk of glioma. Several biological mechanisms may contribute to the observed protective association. EGFR undergoes extensive alternative splicing, generating receptor isoforms with distinct functional properties. Certain soluble isoforms lack the intracellular tyrosine kinase domain and may act as decoy receptors that sequester ligands or interfere with receptor dimerization, thereby attenuating EGFR signaling (Abou-Fayçal et al., 2017; Vorlová et al., 2011). Differential expression of such regulatory isoforms could therefore contribute to the association observed in our MR analysis. In addition, sustained receptor tyrosine kinase signaling can induce negative feedback regulators such as LRIG1 and MIG6 (ERRFI1), which limit receptor activation and promote receptor degradation. These feedback mechanisms may restrain excessive pathway activity and influence tumor-microenvironment interactions (Gur et al., 2004; Yi et al., 2025). Moreover, the genetic instruments used in MR likely reflect overall EGFR expression, whereas oncogenic signaling in glioma is primarily driven by activated and phosphorylated receptor populations resulting from somatic alterations. Consequently, our genetic findings do not contradict the well-established oncogenic driver function of activated EGFR in glioma but instead highlight its biological importance and provide a rationale for our subsequent *in silico* screening of EGFR inhibitors.

Although EGFR inhibitors and monoclonal antibodies have transformed cancer therapy, their application in glioma is often hindered by the BBB, drug resistance, and systemic toxicity (Singh et al., 2025; Taylor et al., 2012). The reference drug in our study, TAK-285, is a potent HER2/EGFR inhibitor capable of crossing the BBB. However, clinical trials have reported dose-limiting toxicities, including hepatotoxicity and adverse events related to non-selective inhibition in normal tissues (Doi et al., 2012). Our study highlights zeylenol and tremulacin as promising alternatives. Unlike the reference synthetic inhibitor TAK-285, which showed potential hepatotoxicity risks in our ADME/TOPKAT analyses, zeylenol exhibited an excellent safety profile with favorable predicted safety profile. Natural products have historically served as the foundation for CNS drugs due to their structural diversity and ability to interact with multiple targets (Bharate and Lindsley, 2024). Similar to other natural EGFR inhibitors like curcumin and scutellarin, which have shown promise in preclinical models (Akter et al., 2021), zeylenol appears to offer a favorable therapeutic window, potentially allowing for the higher dosing required to achieve effective CNS concentrations.

Our computational and biophysical data provide a solid mechanistic explanation for the efficacy of zeylenol. Molecular docking and MD simulations revealed that zeylenol occupies the ATP-binding pocket of EGFR with high stability. Notably, zeylenol established hydrogen bonds with MET793. This interaction mirrors the binding mode of first-generation EGFR-TKIs, such as gefitinib (Yun et al., 2007), which anchor to MET793 within the ATP-binding pocket to reversibly compete with ATP. Furthermore, our screening also identified tremulacin, which formed hydrogen bonds with CYS797. This residue is of particular clinical significance, as the C797S mutation is a known mechanism of resistance to third-generation EGFR inhibitors like osimertinib in other cancers (Wang et al., 2016). The ability of our natural library to identify compounds interacting with these critical residues suggests that this pipeline could potentially be used to screen for inhibitors that overcome specific resistance mutations in the future. The BLI-derived KD of 4.77 μM confirms a direct physical interaction, validating the *in silico* predictions.

Experimental validation in LN18 glioma cells substantiated the therapeutic potential of zeylenol. Zeylenol is a natural product structurally related to zeylenone, a cyclohexene oxide compound originally isolated from the leaves of Uvaria grandiflora (Yang et al., 2018). Notably, zeylenone has demonstrated robust anti-tumor activities against cancer cells in cervical carcinoma, gastric cancer, and prostate cancer (Yang et al., 2018; Zhang et al., 2017; Zeng et al., 2018). Recent study revealed that zeylenone analogs showed potent anti-tumor agent against GBM cells (Su et al., 2023). These findings suggest that compounds within this structural family may represent promising candidates for anticancer drug development, which is consistent with our observation that zeylenol exhibits potent anti-glioma activity.

Despite the promising findings, several limitations warrant consideration. First, our MR analysis relied primarily on datasets of European ancestry; thus, the findings may not be fully generalizable to other populations. While we demonstrated anti-glioma activity in LN18 cells, the therapeutic efficacy, safety profile, and BBB permeability of zeylenol require further verification *in vivo*.

## Data Availability

The original contributions presented in the study are included in the article/Supplementary Material, further inquiries can be directed to the corresponding author.

## References

[B1] Abou-Fayçal C. Hatat A.-S. Gazzeri S. Eymin B. (2017). Splice variants of the RTK family: their role in tumour progression and response to targeted therapy. Int. J. Mol. Sci. 18, 383. 10.3390/ijms18020383 28208660 PMC5343918

[B2] Akter R. Afrose A. Rahman M. R. Chowdhury R. Nirzhor S. S. R. Khan R. I. (2021). A comprehensive analysis into the therapeutic application of natural products as SIRT6 modulators in Alzheimer’s disease, aging, cancer, inflammation, and diabetes. Int. J. Mol. Sci. 22, 4180. 10.3390/ijms22084180 33920726 PMC8073883

[B3] An Z. Aksoy O. Zheng T. Fan Q.-W. Weiss W. A. (2018). Epidermal growth factor receptor and EGFRvIII in glioblastoma: signaling pathways and targeted therapies. Oncogene 37, 1561–1575. 10.1038/s41388-017-0045-7 29321659 PMC5860944

[B4] Bharate S. B. Lindsley C. W. (2024). Natural products driven medicinal chemistry. J. Med. Chem. 67, 20723–20730. 10.1021/acs.jmedchem.4c02736 39629819

[B5] Burgess S. Thompson S. G. (2017). Interpreting findings from Mendelian randomization using the MR-Egger method. Eur. J. Epidemiol. 32, 377–389. 10.1007/s10654-017-0255-x 28527048 PMC5506233

[B7] Colquhoun A. (2017). Cell biology-metabolic crosstalk in glioma. Int. J. Biochem. Cell. Biol. 89, 171–181. 10.1016/j.biocel.2017.05.022 28549626

[B8] Desai M. Di R. Fan H. (2019). Application of biolayer interferometry (BLI) for studying protein-protein interactions in transcription. J. Vis. Exp. (147), e59687. 10.3791/59687 31403627 PMC6693641

[B9] Deschênes-Simard X. Kottakis F. Meloche S. Ferbeyre G. (2014). ERKs in cancer: friends or foes? Cancer Res. 74, 412–419. 10.1158/0008-5472.CAN-13-2381 24408923

[B10] Doi T. Takiuchi H. Ohtsu A. Fuse N. Goto M. Yoshida M. (2012). Phase I first-in-human study of TAK-285, a novel investigational dual HER2/EGFR inhibitor, in cancer patients. Br. J. Cancer 106, 666–672. 10.1038/bjc.2011.590 22240796 PMC3322948

[B11] Ferkingstad E. Sulem P. Atlason B. A. Sveinbjornsson G. Magnusson M. I. Styrmisdottir E. L. (2021). Large-scale integration of the plasma proteome with genetics and disease. Nat. Genet. 53, 1712–1721. 10.1038/s41588-021-00978-w 34857953

[B12] Finan C. Gaulton A. Kruger F. A. Lumbers R. T. Shah T. Engmann J. (2017). The druggable genome and support for target identification and validation in drug development. Sci. Transl. Med. 9, eaag1166. 10.1126/scitranslmed.aag1166 28356508 PMC6321762

[B13] Gaziano L. Giambartolomei C. Pereira A. C. Gaulton A. Posner D. C. Swanson S. A. (2021). Actionable druggable genome-wide Mendelian randomization identifies repurposing opportunities for COVID-19. Nat. Med. 27, 668–676. 10.1038/s41591-021-01310-z 33837377 PMC7612986

[B14] Gur G. Rubin C. Katz M. Amit I. Citri A. Nilsson J. (2004). LRIG1 restricts growth factor signaling by enhancing receptor ubiquitylation and degradation. EMBO J. 23, 3270–3281. 10.1038/sj.emboj.7600342 15282549 PMC514515

[B15] Hemani G. Zheng J. Elsworth B. Wade K. H. Haberland V. Baird D. (2018). The MR-Base platform supports systematic causal inference across the human phenome. Elife 7, e34408. 10.7554/eLife.34408 29846171 PMC5976434

[B16] Irwin J. J. Shoichet B. K. (2005). ZINC--a free database of commercially available compounds for virtual screening. J. Chem. Inf. Model. 45, 177–182. 10.1021/ci049714+ 15667143 PMC1360656

[B17] Katharine Holloway M. McGaughey G. B. Coburn C. A. Stachel S. J. Jones K. G. Stanton E. L. (2007). Evaluating scoring functions for docking and designing beta-secretase inhibitors. Bioorg. Med. Chem. Lett. 17, 823–827. 10.1016/j.bmcl.2006.10.051 17107793

[B18] Lionta E. Spyrou G. Vassilatis D. K. Cournia Z. (2014). Structure-based virtual screening for drug discovery: principles, applications and recent advances. Curr. Top. Med. Chem. 14, 1923–1938. 10.2174/1568026614666140929124445 25262799 PMC4443793

[B19] Liu X. Guo C. Leng T. Fan Z. Mai J. Chen J. (2023). Differential regulation of H3K9/H3K14 acetylation by small molecules drives neuron-fate-induction of glioma cell. Cell. Death Dis. 14, 142. 10.1038/s41419-023-05611-8 36805688 PMC9941105

[B20] Liu Y. Si M. Qian Y. Liu Y. Wang Z. Zhang T. (2023). Bidirectional mendelian randomization analysis investigating the genetic association between primary breast cancer and colorectal cancer. Front. Immunol. 14, 1260941. 10.3389/fimmu.2023.1260941 38283349 PMC10811019

[B21] McNeill K. A. (2016). Epidemiology of brain tumors. Neurol. Clin. 34, 981–998. 10.1016/j.ncl.2016.06.014 27720005

[B22] Melin B. S. Barnholtz-Sloan J. S. Wrensch M. R. Johansen C. Il'yasova D. Kinnersley B. (2017). Genome-wide association study of glioma subtypes identifies specific differences in genetic susceptibility to glioblastoma and non-glioblastoma tumors. Nat. Genet. 49, 789–794. 10.1038/ng.3823 28346443 PMC5558246

[B23] Norden A. D. Wen P. Y. (2006). Glioma therapy in adults. Neurologist 12, 279–292. 10.1097/01.nrl.0000250928.26044.47 17122724

[B24] Pelloski C. E. Ballman K. V. Furth A. F. Zhang L. Lin E. Sulman E. P. (2007). Epidermal growth factor receptor variant III status defines clinically distinct subtypes of glioblastoma. J. Clin. Oncol. 25, 2288–2294. 10.1200/JCO.2006.08.0705 17538175

[B25] Roskoski R. Jr (2019). Small molecule inhibitors targeting the EGFR/erbB family of protein-tyrosine kinases in human cancers. Pharmacol. Res. 139, 395–411. 10.1016/j.phrs.2018.11.014 30500458

[B26] Singh S. Dey D. Barik D. Mohapatra I. Kim S. Sharma M. (2025). Glioblastoma at the crossroads: current understanding and future therapeutic horizons. Signal Transduct. Target. Ther. 10, 213. 10.1038/s41392-025-02299-4 40628732 PMC12238593

[B27] Stupp R. Mason W. P. van den Bent M. J. Weller M. Fisher B. Taphoorn M. J. B. (2005). Radiotherapy plus concomitant and adjuvant temozolomide for glioblastoma. N. Engl. J. Med. 352, 987–996. 10.1056/NEJMoa043330 15758009

[B28] Su R. Cao W. Ma G. Li W. Li Z. Liu Y. (2023). Cyclohexene oxide CA, a derivative of zeylenone, exhibits anti-cancer activity in glioblastoma by inducing G0/G1 phase arrest through interference with EZH2. Front. Pharmacol. 14, 1326245. 10.3389/fphar.2023.1326245 38264522 PMC10803536

[B29] Sun B. B. Maranville J. C. Peters J. E. Stacey D. Staley J. R. Blackshaw J. (2018). Genomic atlas of the human plasma proteome. Nature 558, 73–79. 10.1038/s41586-018-0175-2 29875488 PMC6697541

[B30] Taylor T. E. Furnari F. B. Cavenee W. K. (2012). Targeting EGFR for treatment of glioblastoma: molecular basis to overcome resistance. Curr. Cancer Drug Targets 12, 197–209. 10.2174/156800912799277557 22268382 PMC3464093

[B31] Vorlová S. Rocco G. Lefave C. V. Jodelka F. M. Hess K. Hastings M. L. (2011). Induction of antagonistic soluble decoy receptor tyrosine kinases by intronic polyA activation. Mol. Cell. 43, 927–939. 10.1016/j.molcel.2011.08.009 21925381 PMC3781938

[B32] Võsa U. Claringbould A. Westra H. J. Bonder M. J. Deelen P. Zeng B. (2021). Large-scale cis- and trans-eQTL analyses identify thousands of genetic loci and polygenic scores that regulate blood gene expression. Nat. Genet. 53, 1300–1310. 10.1038/s41588-021-00913-z 34475573 PMC8432599

[B6] Wang D. Liu S. Warrell J. Won H. Shi X. Navarro F. C. P. (2018). Comprehensive functional genomic resource and integrative model for the human brain. Science 362, eaat8464. 10.1126/science.aat8464 30545857 PMC6413328

[B33] Wang S. Tsui S. T. Liu C. Song Y. Liu D. (2016). EGFR C797S mutation mediates resistance to third-generation inhibitors in T790M-positive non-small cell lung cancer. J. Hematol. Oncol. 9, 59. 10.1186/s13045-016-0290-1 27448564 PMC4957905

[B34] Wu M. Zhang P. (2020). EGFR-Mediated autophagy in tumourigenesis and therapeutic resistance. Cancer Lett. 469, 207–216. 10.1016/j.canlet.2019.10.030 31639425

[B35] Wu G. Robertson D. H. Brooks C. L. 3rd Vieth M. (2003). Detailed analysis of grid-based molecular docking: a case study of CDOCKER-A CHARMm-based MD docking algorithm. J. Comput. Chem. 24, 1549–1562. 10.1002/jcc.10306 12925999

[B36] Yang S. Liao Y. Li L. Xu X. Cao L. (2018). Zeylenone induces mitochondrial apoptosis and inhibits migration and invasion in gastric cancer. Molecules 23, 2149. 10.3390/molecules23092149 30150551 PMC6225419

[B37] Yang W. Wang S. Zhang X. Sun H. Zhang M. Chen H. (2022). New natural compound inhibitors of PDGFRA (platelet-derived growth factor receptor α) based on computational study for high-grade glioma therapy. Front. Neurosci. 16, 1060012. 10.3389/fnins.2022.1060012 36685223 PMC9845622

[B38] Yang W. Ge J. Yuan M. Li J. Pan L. Ren J. (2023). Computational study of novel natural inhibitors targeting kirsten rat sarcoma viral oncogene homolog G12C. Anticancer Drugs 34, 609–619. 10.1097/CAD.0000000000001428 36847041

[B39] Yang X.-Z. Huang M. Y. Han F. Ni J. Zhou L. X. Yao M. (2024). Genome-wide Mendelian randomization study reveals druggable genes for cerebral small vessel disease. Stroke 55, 2264–2273. 10.1161/STROKEAHA.124.046544 39114924

[B40] Ye C. Dou C. Liu D. Kong L. Chen M. Xu M. (2025). Multivariate genome-wide analyses of insulin resistance unravel novel loci and therapeutic targets for cardiometabolic health. Nat. Commun. 16, 10057. 10.1038/s41467-025-64985-9 41249132 PMC12623863

[B41] Yi S. A. Cho D. Kim S. Kim H. Choi M. K. Choi H. S. (2025). Functional loss of ERBB receptor feedback inhibitor 1 (MIG6) promotes glioblastoma tumorigenesis by aberrant activation of epidermal growth factor receptor (EGFR). Mol. Oncol. 19, 937–953. 10.1002/1878-0261.13717 39129344 PMC11887669

[B42] Yun C.-H. Boggon T. J. Li Y. Woo M. S. Greulich H. Meyerson M. (2007). Structures of lung cancer-derived EGFR mutants and inhibitor complexes: mechanism of activation and insights into differential inhibitor sensitivity. Cancer Cell. 11, 217–227. 10.1016/j.ccr.2006.12.017 17349580 PMC1939942

[B43] Zeng S. Zhu B. Zeng J. Wu W. Jiang C. (2018). Zeylenone represses the progress of human prostate cancer by downregulating the Wnt/β-catenin pathway. Mol. Med. Rep. 18, 5572–5578. 10.3892/mmr.2018.9564 30365080 PMC6236222

[B44] Zhang L. Huo X. Liao Y. Yang F. Gao L. Cao L. (2017). Zeylenone, a naturally occurring cyclohexene oxide, inhibits proliferation and induces apoptosis in cervical carcinoma cells *via* PI3K/AKT/mTOR and MAPK/ERK pathways. Sci. Rep. 7, 1669. 10.1038/s41598-017-01804-2 28490807 PMC5431878

[B45] Zhang A. Guo Z. Ren J. X. Chen H. Yang W. Zhou Y. (2023). Development of an MCL-1-related prognostic signature and inhibitors screening for glioblastoma. Front. Pharmacol. 14, 1162540. 10.3389/fphar.2023.1162540 37538176 PMC10394558

[B46] Zhao N. Zhang J. Zhao Q. Chen C. Wang H. (2021). Mechanisms of long non-coding RNAs in biological characteristics and aerobic glycolysis of glioma. Int. J. Mol. Sci. 22, 11197. 10.3390/ijms222011197 34681857 PMC8541290

[B47] Zhao Y. Li W. Zhang K. Xu M. Zou Y. Qiu X. (2022). Revealing oxidative stress-related genes in osteoporosis and advanced structural biological study for novel natural material discovery regarding MAPKAPK2. Front. Endocrinol. (Lausanne) 13, 1052721. 10.3389/fendo.2022.1052721 36479222 PMC9720258

[B48] Zhong S. Wu B. Yang W. Ge J. Zhang X. Chen Z. (2021). Effective natural inhibitors targeting poly ADP-ribose polymerase by computational study. Aging (Albany NY) 13, 1898–1912. 10.18632/aging.103986 33486472 PMC7880371

[B49] Zhong S. Zhang Z. Guo Z. Yang W. Dou G. Lv X. (2022). Identification of novel natural inhibitors targeting AKT serine/threonine kinase 1 (AKT1) by computational study. Bioengineered 13, 12003–12020. 10.1080/21655979.2021.2011631 35603567 PMC9275969

[B50] Zhong S. Yang W. Zhang Z. Xie Y. Pan L. Ren J. (2023). Association between viral infections and glioma risk: a two-sample bidirectional Mendelian randomization analysis. BMC Med. 21, 487. 10.1186/s12916-023-03142-9 38053181 PMC10698979

